# The 100 most cited papers on total anomalous pulmonary venous connection: a bibliometric analysis

**DOI:** 10.1186/s13019-023-02284-4

**Published:** 2023-05-25

**Authors:** Chen Wen, Wei Liu, Chenhao Fang, Jin Shentu, Ruixiang Ma, Han Zhang, Hao Zhang, Zhongqun Zhu, Huiwen Chen

**Affiliations:** 1 Department of Cardiothoracic Surgery, Shanghai Children’s Medical Center, School of Medicine, Shanghai Jiao Tong University, Shanghai, China; 2 Department of Neurosurgery, Shanghai Children’s Medical Center, School of Medicine, Shanghai Jiao Tong University, Shanghai, China

**Keywords:** Total anomalous pulmonary venous connection, Citation analysis, Bibliometrics analysis, Citation classics

## Abstract

**Background:**

The number of citations a paper receives reflects its impact on the scientific community. We aimed to identify and explore the characteristics of the most cited papers on total anomalous pulmonary venous connection (TAPVC).

**Methods:**

Web of Science Core Collection Expanded Science Citation Index (1900 to present) was searched and papers on TAPVC were reviewed. Articles were ranked by the number of citations and the 100 most cited papers were analyzed.

**Results:**

The 100 most cited papers were published between 1952 and 2018 with a mean number of citations of 52 (range 26 to 148). The 1990s was the most productive decade. All articles except one were written in English. The 100 most cited articles were published in 24 journals, led by Journal of Thoracic and Cardiovascular Surgery (21 articles), followed by Annals of Thoracic Surgery (20 articles), and Circulation (16 articles). The United States of America contributed most of the 100 most cited papers (60 articles). Hospital for Sick Children, Toronto led the list of citation classics with six papers. Christopher A. Caldarone, John W. Kirklin, and P. E. F. Daubeney were the most productive authors with 3 articles each. More than half of the papers were cohort studies (51 articles). Surgery, radiology and etiology were the main topics. Thirty-one articles were funded by public foundations, and none received support from commercial companies.

**Conclusions:**

The bibliometric analysis gives a historical perspective on scientific progress in the field of TAPVC and lays the foundation for future research.

**Supplementary Information:**

The online version contains supplementary material available at 10.1186/s13019-023-02284-4.

## Background

Total anomalous pulmonary venous connection (TAPVC) is a rare congenital anomaly, constituting ≈ 1–3% of congenital heart disease, where none of the pulmonary veins connects normally to the left atrium [1]. Despite progress in surgical intervention, it remains to be associated with disappointing prognosis [2, 3]. Experts and researchers try their best to explore the pathogenesis, diagnosis and management of TAPVC, and a large number of literatures about TAPVC has been published over time. It is important to get a better understanding of the progression in the field of TAPVC and its impact on clinical practice. Among published papers, the classic papers have greatly contributed to the development of the field of TAPVC. However, how to identify these important articles remains to be resolved.

There are many ways to assess the importance of a paper. Bibliometrics uses quantitative methods like citation analysis to evaluate the impact of a literature on the scientific community. Citation analysis as a popular tool is used to measure the recognition of a paper based on the number of citations it receives. The rational is that important papers will be cited more frequently. Although drawbacks exist when evaluating the impact of a paper based only on the number of citations, it is currently the most accepted approach. The citation analysis of the most cited papers has been widespread in different research areas. However, a bibliometric analysis of the most cited papers on TAPVC is not available. The aim of this study was to identify and explore the characteristics of the 100 most cited papers on TAPVC.

## Methods

The database of the Clarivate Analytics’ Web of Science Core Collection Expanded Science Citation Index (1900 to present) was searched using the topic words “total anomalous pulmonary venous connection”, “total anomalous pulmonary venous drainage” or “total anomalous pulmonary venous return” on 21 May 2022. This database indexed more than 53 million records from over 9500 high impact journals across 178 scientific disciplines. The ‘document type’ was activated to limit the format of papers to ‘article’ or ‘review’. Articles retrieved were ranked by the number of citations, from highest to lowest. Two authors read the abstract or full text if needed to identify the 100 most cited papers relevant to TAPVC. The following information was recorded: title, author names, the number of citations, publication year, country origin, institution, journal, funding source, and article type or subspecialty. Disagreements between authors was resolved by consensus. The institution and country origin were defined by the address of the corresponding author. The Pearson’s test was used to determine the correlation between two continuous variables. The data of gross domestic product (GDP) per capita were retrieved from the World Bank (www.worldbank.org). Statistical analysis was performed using SPSS version 24.0 (SPSS Inc., Chicago, IL, USA). P < 0.05 was considered statistically significant.

## Results

A total of 1352 papers were identified after the initial search, with 984 as ‘article’ and 32 as ‘review’. The 100 most cited papers (Additional file 1) were published between 1952 and 2018 with a mean number of citations of 52 (range 26–148), and 7 papers were cited more than 100 times.

The decade 1990 to 1999 produced the most citation classics with 21 articles followed by the decades from 1970 to 1979 and 2010 to 2019 with 18 articles each (Table 1). The most citation classics published within a given year were 4 articles each in 1956, 1978, 1992, 1996, 2010, 2012 and 2013. More than 80% of the papers were published after 1970 (84 articles).

**Table 1:** Frequency distribution showing publication years of the 100 most cited articles

Decade	Number of articles
1950–1959	7
1960–1969	9
1970–1979	18
1980–1989	12
1990–1999	21
2000–2009	15
2010–2019	18

The 100 most cited papers were published in 24 journals, predominantly in Journal of Thoracic and Cardiovascular Surgery (21 articles), followed by Annals of Thoracic Surgery (20 articles) and Circulation (16 articles) (Table 2). Eleven journals indexed more than one citation classic. There was no correlation between journal impact factor and the number of 100 most cited papers (r = 0.048, P = 0.824) or the number of citations (r = 0.142, P = 0.160).

**Table 2:** Frequent journals of the 100 most cited papers (Included if ≥ 2 articles were in top cited list)

Journal	Impact factor (2020)	Number of articles
Journal of Thoracic and Cardiovascular Surgery	5.209	21
Annals of Thoracic Surgery	4.330	20
Circulation	29.69	16
British Heart Journal/Heart	5.994	7
European Journal of Cardio-Thoracic Surgery	4.191	6
Proceedings of the Staff Meetings of the Mayo Clinic/Mayo Clinic Proceedings	7.619	3
Ultrasound in Obstetrics & Gynecology	7.299	3
American Heart Journal	4.749	3
American Journal of Cardiology	2.778	3
American Journal of Roentgenology Radium Therapy and Nuclear Medicine/American Journal of Roentgenology	3.959	3
Surgery Gynecology & Obstetrics/Journal of the American College of Surgeons	6.113	2

The 100 cited articles originated from 13 countries, with the United States of America (USA) (60 articles), United Kingdom (11 articles) and Canada (8 articles) being the most prolific (Table 3). Ten countries contributed more than one citation classic. Only 18 papers originated from non-English speaking countries. There was no correlation between the GDP per capita and the numbers of 100 most cited papers on TAPVC (r = 0.452, P = 0.121).

**Table 3:** Frequent countries of the 100 most cited papers (Included if ≥ 2 articles were in most cited list)

Country	Number of articles
United States of America	60
United Kingdom	11
Canada	8
France	4
Italy	3
Japan	3
Australia	2
China	2
India	2
South Korea	2

Eighteen institutions produced more than one citation classic (Table 4). Hospital For Sick Children, Toronto was found to be the most productive institution with 6 articles, followed by Boston Children’s Hospital, Children’s Memorial Hospital, Mayo Clinic and Mayo Foundation, Texas Children’s Hospital, and Royal Brompton Hospital with 5 articles each.

**Table 4:** Frequent institutions of the 100 most cited papers (Included if ≥ 2 articles were in most cited list)

Institution	Number of articles
Hospital For Sick Children, Toronto	6
Boston Children’s Hospital	5
Children’s Memorial Hospital	5
Mayo Clinic and Mayo Foundation	5
Texas Children’s Hospital	5
Royal Brompton Hospital	5
Children’s Hospital of Philadelphia	4
Great Ormond Street Hospital for Children	4
Marie-Lannelongue Hospital	3
St. Luke’s Episcopal Hospital	3
All India Institute of Medical Sciences	2
Indiana University Medical Center	2
Royal Children’s Hospital	2
St. Luke’s Episcopal Hospital	2
The Children’s Hospital of Iowa at the University of Iowa Hospitals and Clinics	2
University of Utah School of Medicine	2
University of Michigan Hospital	2
Columbia University, the Babies Hospital	2

The number of authors of the most cited papers ranged from one to 16. Table 5 presents a list of the most productive authors with two or more citation classics. Christopher A. Caldarone, John W. Kirklin, and P. E. F. Daubeney were the most productive authors with 3 articles each.

**Table 5:** Frequent authors of the 100 most cited papers (Included if ≥ 2 articles were in most cited list)

Corresponding author	Number of articles
Christopher A. Caldarone	3
John W. Kirklin	3
P. E. F. Daubeney	3
David L. S. Morales	2
Denton A. Cooley	2
Edwards, JE	2
Francois Lacour-Gayet	2
J. William Gaynor	2
Richard Van Praagh	2
Ujjwal K. Chowdhury	2

The majority of the 100 most cited papers were clinical articles (95 articles), with the remaining basic science (5 articles) (Table 6). Of 95 clinical papers, cohort study (51 articles) was the most common type, and only 1 reported a clinical trial. Surgery (70 articles) is the main topic of the most cited papers, followed by radiology (17 articles), and etiology (6 articles). Among these articles, 31 were funded by public foundations, none received support from commercial companies, for the remaining 69 the funding source was not specified. Specifically, 15 studies received grants from the National Institutes of Health.

**Table 6:** Study type of the 100 most cited papers

Study type	Number of articles
Clinical trial	1
Cohort study	51
Case-control study	1
Case series	33
Case report	6
Review article	3
Basic science	5

## Discussion

In this study, we identified the most cited papers on TAPVC and also summarized the characteristics of highly cited articles. The lists of the most cited papers demonstrated the history and development on TAPVC. To our knowledge, this is the first study on the most cited articles in the field of TAPVC. This list of 100 most cited papers is of substantial importance for several reasons. Authors can learn about the research hotspots and shape their research interests. This list can serve as an education tool for residents and directors so that they can be familiar with the classic papers on TAPVC [4]. Journal editors and reviewers can identify potential highly cited papers with a prior knowledge of typical characteristics of such papers when they evaluate submitted manuscripts. Clinicians can apply important findings to the clinical practice, therefore patients may obtain better outcomes.

The most cited paper on the list, which described 93 autopsied cases with TAPVC, was written by Delisle et al. in 1976 [5].This manuscript found that TAPVC was failure of development of the common pulmonary vein with collaterals between the lungs and the systemic veins. TAPVC was a rapidly fatal disease and the age at death was influenced by obstruction of the anomalous venous pathway. TAPVC could almost always be corrected by surgery according to the anatomy. This paper emphasized diagnostic and surgical considerations in TAPVC. The second most cited article, which presented the clinical and physiologic observations of 75 pediatric patients with TAPVC, was written by Gathman et al. in 1970 [6].The authors proposed an approach to managing TAPVC. Patients with pulmonary venous obstruction should undergo early surgery. Patients with pulmonary hypertension without pulmonary venous obstruction should receive medical intervention. If pulmonary hypertension was not present and particularly if there was a significant gradient across the right ventricular out flow tract, medical treatment might to be recommended until operation could be performed.

The oldest paper on the list, which described three patients with TAPVC, was written by Parsons et al. in 1952 [7].At that time, the anastomosis of pulmonary veins to the appendage was feasible only in the experimental animal. The authors proposed that it should be possible to correct anomalous pulmonary veins by transplantation of pulmonary veins in man. This paper laid the foundation for the surgical repair of TAPVC. The latest paper on the list, which explored the potential genetic abnormalities in patients with TAPVC through next-generation sequencing, was written by Shi et al. in 2018 [8]. This study identified SNAI1, HMGA2 and VAV2 as novel candidate genes associated with TAPVC and provided novel insights into pulmonary vein development. This paper is helpful to understand the pathogenesis of TAPVC.

Interestingly, one of the 100 most cited papers which ranked 21 was published by our team in Circulation. [9] This multi-institutional study used a large cohort of patients (768 patients) to assess the impact of current management strategies on the outcomes of TAPVC. This study found that contemporary outcomes after surgical repair for TAPVC were acceptable. This study examined risk factors associated with outcomes and suggested that computed tomography angiography plays an important role in the preoperative morphological evaluation of TAPVC and is useful in preoperative surgical planning. This study also found that compared with the conventional repair, the sutureless technique could reduce the restenosis rate in patients with preoperative pulmonary venous obstruction (PVO) and that no statistical difference was found in patients without preoperative PVO. Our team is dedicated to the study of TAPVC and has published some related articles recently.

Compared with bibliometric analyses in other specialties, the number of citations of the 100 most cited papers on TAPVC is much lower. The highest cited paper in a bibliometric analysis on mitral valve surgery received 1348 citations with a mean (range) citation of 248 (148–1348). [10] The highest cited paper in a bibliometric analysis on minimally-invasive cardiac surgery received 414 citations with a median (range) citation of 101 (51–414). [11] It might indicate that a lower degree of research activity within TAPVC in comparison to more established fields.

Our analysis found that the 100 most cited articles were published in 24 journals. A total of 79 articles were published in only 10 cardiovascular specialty journals. This shows a trend of publishing influential papers in specialty journals compared with non-specialty journals. Our findings supported the application of Bradford’s law, a bibliometric concept put forward by Brookes and Siegelman [12, 13]. The main idea behind Bradford’s law is that researchers obtain citations from a few core journals in their specialized field. When researchers deviate from core journals, the citations and impact fall. This trend makes most highly cited papers come from a few professional journals.

Considering the fact that the journal impact factor is calculated based on the number of citations, the journal impact factor should be positively correlated with the status of citations. But the results of previous bibliometric papers on different specialties have not been consistent in this relationship. Some studies demonstrated that the journal impact factor was positively correlated with the number of the most cited papers and citations [14], while some others did not [11]. The correlation between journal impact factor and the status of citations on TAPVC was weak in our study. Our study did not support the well-known law that the most cited articles are often published in journals with high impact factor, which in turn contribute to the high impact factor of these journals. The possible reason is that compared with other specialities, TAPVC is still a very new field of interest and currently in its infancy. The weak relationship may be due to a lack of time, and will strengthen as the field develops and time passes.

All the 100 articles except one were published in English and only 18 papers originated from non-English speaking countries. It demonstrated the dominance of English in the scientific community. The majority of highly cited papers were from the USA. This phenomenon also exists in other disciplines which indicates the leadership of the USA in the scientific community. It may be due to the large population of researchers and sufficient financial support for scientific research in USA. However, there could be many other potential factors that lead to the dominance of USA. There is a tendency that American authors cite native papers and American journals accept papers from native authors [15, 16]. It is generally accepted that scientific research output depends on the national economy [17]. But the results of previous bibliometric papers on different specialties have not been consistent in this relationship. Some bibliometric papers found a significantly positive correlation between GDP per capita and the status of citations [18], while some other bibliometric papers could not [19]. The correlation between the GDP per capita and the number of the 100 most cited papers on TAPVC was weak in our study. This may be explained by the limited number of papers and countries, government policy, the level of research and medical infrastructure, research funding, the culture around research, research and development, the number of universities and research institutions [20].

Clinical research bridges the gap between basic science and clinical practice. Our analysis demonstrated that the vast majority of papers on TAPVC were clinical studies. Similar with previous studies, there is lack of papers with high level of evidence. Randomized controlled trial (RCT) tops the evidence pyramid and facilitates the application of research findings to clinical practice decisions [21]. However, RCT-based study does not exist on TAPVC. The likely reason is that TAPVC can only be treated with surgical repair and RCT in surgery may be difficult to achieve. Another reason may be that new findings are usually initially published as observational studies, and still receive the attention of the researchers. The list of the most cited papers shows some hot topics in TAPVC research. Surgery is the most popular topic in the list as surgical repair is the only effective treatment. Diagnosis of TAPVC remains controversial. Therefore, radiology is another hot topic in the field of TAPVC.

Fund support is a great boost to medical research. In our study, 31 papers reported funding support from public foundations. However, our review showed that no research projects received grants from commercial resource. Although commercial support is debated because of the susceptibility to various kinds of biases, it has played a critical role in the research process. A remarkable thing should be considered that some old papers might not have fully reported their financial conflicts.

We also demonstrated that the majority (84 papers) of the most cited articles were published after 1970. Our finding is also verified in other bibliometric analyses [4, 14]. The body of literature has flourished tremendously and great progress has been made in recent years in the field of TAPVC, and clinicians tend to rely on the latest literatures, which may explain this phenomenon. There were no papers published before 1950 in the list which indicates that old papers have limited value in modern times. However, it is important to realize that this trend could be influenced by multiple issues. Limitations in the database for indexing older literatures, lack of online resources, and the tendency to publish papers in the form of textbooks in the early times may contribute to this trend.

Although we have tried to reduce potential biases, some limitations were inevitable and inherent to citation analysis. First, citation analysis is not a perfect method to measure the impact of an article on the scientific community. Citation analysis is not a measure of the quality of an article, but rather the recognition. In other words, the number of citations an article receives does not accurately reflect its importance. Second, The overall number of citations an article is cited used as a measure of impact will favor older articles and omit important articles from the past 10 years [22]. The number of citations an article receives depends on its publication year, since citations accrue over time. Third, there is a snowball effect in citations due to adherence to scientific paradigms [23]. Some authors tend to cite certain articles, simply because they have been cited for many times rather than for their importance. Fourth, the bibliometric phenomenon, termed “obliteration by incorporation” is also a confounder. Some pioneering studies are assimilated into current knowledge and no longer cited. These important works may be underestimated with respect to the impact on the scientific community. Fifth, authors do not necessarily cite the articles that have influenced them most. Citing is a complex process with many motives and are strongly affected by self-citation, in-house citation, citing high-impact journals and review articles, and national or language preferences [24, 25]. Last, we performed the citation analysis with only one of the many available databases. No database is considered superior, and the number of citations an article receives varies greatly across different databases [26–28]. It is possible that many articles that were not included on our list may be included if we use other databases for citation analysis.

## Conclusions

In conclusion, the bibliometric analysis gives a historical perspective on the scientific progress in the field of TAPVC and lays the foundation for future research. This study presents a detailed list and analysis of the 100 most cited articles on TAPVC. The most cited papers on the list were all except one published in English, and were mainly cohort studies. Most of the articles were published in Journal of Thoracic and Cardiovascular Surgery and Annals of Thoracic Surgery and originated from USA. The present study may help to find out the important factors that contribute to citation classics on TAPVC.

## Electronic supplementary material

Below is the link to the electronic supplementary material.

**Additional File 1:** Supplemental Material

## Data Availability

The datasets used and/or analysed during the current study are available from the corresponding author on reasonable request.

## Abbreviations

GDP: gross domestic product
PVO: pulmonary venous obstruction
RCT: randomized controlled trial
TAPVC: total anomalous pulmonary venous connection
USA: United States of America
