# Supplementary material for: The 100 most cited papers on total anomalous pulmonary venous connection: a bibliometric analysis
Source: J Cardiothorac Surg. 2023 May 25;18:187. doi: 10.1186/s13019-023-02284-4 (PMC10214610; doi:10.1186/s13019-023-02284-4)
Supplement: Supplementary file 1 — Additional File 1: Supplemental Material [file 13019_2023_2284_MOESM1_ESM.docx]

**SUPPLEMENTAL MATERIAL**

**Table S1** The 100 most cited papers on total anomalous pulmonary venous connection

| **Rank** | **Publication** | **Citations** |
| --- | --- | --- |
| 1 | Delisle G, Ando M, Calder AL, Zuberbuhler JR, Rochenmacher S, Alday LE, et al. Total anomalous pulmonary venous connection: Report of 93 autopsied cases with emphasis on diagnostic and surgical considerations. Am Heart J. 1976;91(1):99-122. | 148 |
| 2 | Gathman GE, Nadas AS. Total anomalous pulmonary venous connection: clinical and physiologic observations of 75 pediatric patients. Circulation. 1970;42(1):143-54. | 139 |
| 3 | Atz AM, Adatia I, Wessel DL. Rebound pulmonary hypertension after inhalation of nitric oxide. Ann Thorac Surg. 1996;62(6):1759-64. | 137 |
| 4 | Karamlou T, Gurofsky R, Al Sukhni E, Coles JG, Williams WG, Caldarone CA, et al. Factors associated with mortality and reoperation in 377 children with total anomalous pulmonary venous connection. Circulation. 2007;115(12):1591-8. | 131 |
| 5 | Hancock Friesen CL, Zurakowski D, Thiagarajan RR, Forbess JM, del Nido PJ, Mayer JE, et al. Total anomalous pulmonary venous connection: an analysis of current management strategies in a single institution. Ann Thorac Surg. 2005;79(2):596-606; discussion 596-606. | 129 |
| 6 | Burroughs JT, Edwards JE. Total anomalous pulmonary venous connection. Am Heart J. 1960;59:913-31. | 129 |
| 7 | Seale AN, Uemura H, Webber SA, Partridge J, Roughton M, Ho SY, et al. Total anomalous pulmonary venous connection: morphology and outcome from an international population-based study. Circulation. 2010;122(25):2718-26. | 116 |
| 8 | Caldarone CA, Najm HK, Kadletz M, Smallhorn JF, Freedom RM, Williams WG, et al. Relentless pulmonary vein stenosis after repair of total anomalous pulmonary venous drainage. Ann Thorac Surg. 1998;66(5):1514-20. | 84 |
| 9 | Gaynor JW, Collins MH, Rychik J, Gaughan JP, Spray TL. Long-term outcome of infants with single ventricle and total anomalous pulmonary venous connection. J Thorac Cardiovasc Surg. 1999;117(3):506-13; discussion 13-4. | 83 |
| 10 | Lacour-Gayet F, Zoghbi J, Serraf AE, Belli E, Piot D, Rey C, et al. Surgical management of progressive pulmonary venous obstruction after repair of total anomalous pulmonary venous connection. J Thorac Cardiovasc Surg. 1999;117(4):679-87. | 78 |
| 11 | Lewis FJ, Varco RL, Taufic M, Niazi SA. Direct vision repair of triatrial heart and total anomalous pulmonary venous drainage. Surg Gynecol Obstet. 1956;102(6):713-20. | 77 |
| 12 | Katz NM, Kirklin JW, Pacifico AD. Concepts and practices in surgery for total anomalous pulmonary venous connection. Ann Thorac Surg. 1978;25(5):479-87. | 75 |
| 13 | Cooley DA, Hallman GL, Leachman RD. Total anomalous pulmonary venous drainage; correction with the use of cardiopulmonary bypass in 62 cases. J Thorac Cardiovasc Surg. 1966;51(1):88-102. | 71 |
| 14 | Kelle AM, Backer CL, Gossett JG, Kaushal S, Mavroudis C. Total anomalous pulmonary venous connection: results of surgical repair of 100 patients at a single institution. J Thorac Cardiovasc Surg. 2010;139(6):1387-94 e3. | 69 |
| 15 | Hyde JA, Stumper O, Barth MJ, Wright JG, Silove ED, de Giovanni JV, et al. Total anomalous pulmonary venous connection: outcome of surgical correction and management of recurrent venous obstruction. Eur J Cardiothorac Surg. 1999;15(6):735-40; discussion 40-1. | 69 |
| 16 | Turley K, Tucker WY, Ullyot DJ, Ebert PA. Total anomalous pulmonary venous connection in infancy: influence of age and type of lesion. Am J Cardiol. 1980;45(1):92-7. | 69 |
| 17 | Hastreiter AR, Paul MH, Molthan ME, Miller RA. Total anomalous pulmonary venous connection with severe pulmonary venous obstruction. A clinical entity. Circulation. 1962;25:916-28. | 69 |
| 18 | Cooley DA, Ochsner A, Jr. Correction of total anomalous pulmonary venous drainage: technical considerations. Surgery. 1957;42(6):1014-21. | 69 |
| 19 | Bando K, Turrentine MW, Ensing GJ, Sun K, Sharp TG, Sekine Y, et al. Surgical management of total anomalous pulmonary venous connection. Thirty-year trends. Circulation. 1996;94(9 Suppl):II12-6. | 67 |
| 20 | Sahn DJ, Allen HD, Lange LW, Goldberg SJ. Cross-sectional echocardiographic diagnosis of the sites of total anomalous pulmonary venous drainage. Circulation. 1979;60(6):1317-25. | 67 |
| 21 | Shi G, Zhu Z, Chen J, Ou Y, Hong H, Nie Z, et al. Total Anomalous Pulmonary Venous Connection: The Current Management Strategies in a Pediatric Cohort of 768 Patients. Circulation. 2017;135(1):48-58. | 66 |
| 22 | Allan LD, Sharland GK. The echocardiographic diagnosis of totally anomalous pulmonary venous connection in the fetus. Heart. 2001;85(4):433-7. | 65 |
| 23 | Correa-Villasenor A, Ferencz C, Boughman JA, Neill CA. Total anomalous pulmonary venous return: familial and environmental factors. The Baltimore-Washington Infant Study Group. Teratology. 1991;44(4):415-28. | 64 |
| 24 | Ricci M, Elliott M, Cohen GA, Catalan G, Stark J, de Leval MR, et al. Management of pulmonary venous obstruction after correction of TAPVC: risk factors for adverse outcome. Eur J Cardiothorac Surg. 2003;24(1):28-36; discussion. | 63 |
| 25 | Paquet M, Gutgesell H. Echocardiographic features of total anomalous pulmonary venous connection. Circulation. 1975;51(4):599-605. | 63 |
| 26 | Kim TH, Kim YM, Suh CH, Cho DJ, Park IS, Kim WH, et al. Helical CT angiography and three-dimensional reconstruction of total anomalous pulmonary venous connections in neonates and infants. AJR Am J Roentgenol. 2000;175(5):1381-6. | 61 |
| 27 | Kirshbom PM, Myung RJ, Gaynor JW, Ittenbach RF, Paridon SM, DeCampli WM, et al. Preoperative pulmonary venous obstruction affects long-term outcome for survivors of total anomalous pulmonary venous connection repair. Ann Thorac Surg. 2002;74(5):1616-20. | 59 |
| 28 | Sano S, Brawn WJ, Mee RB. Total anomalous pulmonary venous drainage. J Thorac Cardiovasc Surg. 1989;97(6):886-92. | 59 |
| 29 | Lacour-Gayet F, Rey C, Planche C. [Pulmonary vein stenosis. Description of a sutureless surgical procedure using the pericardium in situ]. Arch Mal Coeur Vaiss. 1996;89(5):633-6. | 58 |
| 30 | Haworth SG, Reid L. Structural study of pulmonary circulation and of heart in total anomalous pulmonary venous return in early infancy. Br Heart J. 1977;39(1):80-92. | 55 |
| 31 | Yong MS, d'Udekem Y, Robertson T, Horton S, Dronavalli M, Brizard C, et al. Outcomes of surgery for simple total anomalous pulmonary venous drainage in neonates. Ann Thorac Surg. 2011;91(6):1921-7. | 54 |
| 32 | Kirshbom PM, Flynn TB, Clancy RR, Ittenbach RF, Hartman DM, Paridon SM, et al. Late neurodevelopmental outcome after repair of total anomalous pulmonary venous connection. J Thorac Cardiovasc Surg. 2005;129(5):1091-7. | 54 |
| 33 | Hammon JW, Jr., Bender HW, Jr., Graham TP, Jr., Boucek RJ, Jr., Smith CW, Erath HG, Jr. Total anomalous pulmonary venous connection in infancy. Ten years' experience including studies of postoperative ventricular function. J Thorac Cardiovasc Surg. 1980;80(4):544-51. | 54 |
| 34 | Whight CM, Barratt-Boyes BG, Calder AL, Neutze JM, Brandt PW. Total anomalous pulmonary venous connection. Long-term results following repair in infancy. J Thorac Cardiovasc Surg. 1978;75(1):52-63. | 53 |
| 35 | Degenhardt K, Singh MK, Aghajanian H, Massera D, Wang Q, Li J, et al. Semaphorin 3d signaling defects are associated with anomalous pulmonary venous connections. Nat Med. 2013;19(6):760-5. | 52 |
| 36 | Bleyl S, Nelson L, Odelberg SJ, Ruttenberg HD, Otterud B, Leppert M, et al. A gene for familial total anomalous pulmonary venous return maps to chromosome 4p13-q12. Am J Hum Genet. 1995;56(2):408-15. | 52 |
| 37 | Bleyl SB, Saijoh Y, Bax NA, Gittenberger-de Groot AC, Wisse LJ, Chapman SC, et al. Dysregulation of the PDGFRA gene causes inflow tract anomalies including TAPVR: integrating evidence from human genetics and model organisms. Hum Mol Genet. 2010;19(7):1286-301. | 51 |
| 38 | Wukasch DC, Deutsch M, Reul GJ, Hallman GL, Cooley DA. Total anomalous pulmonary venous return. Review of 125 patients treated surgically. Ann Thorac Surg. 1975;19(6):622-33. | 51 |
| 39 | Yanagawa B, Alghamdi AA, Dragulescu A, Viola N, Al-Radi OO, Mertens LL, et al. Primary sutureless repair for "simple" total anomalous pulmonary venous connection: midterm results in a single institution. J Thorac Cardiovasc Surg. 2011;141(6):1346-54. | 50 |
| 40 | Michielon G, Di Donato RM, Pasquini L, Giannico S, Brancaccio G, Mazzera E, et al. Total anomalous pulmonary venous connection: long-term appraisal with evolving technical solutions. Eur J Cardiothorac Surg. 2002;22(2):184-91. | 49 |
| 41 | Gomes MM, Feldt RH, McGoon DC, Danielson GK. Total anomalous pulmonary venous connection. Surgical considerations and results of operation. J Thorac Cardiovasc Surg. 1970;60(1):116-22. | 49 |
| 42 | Morales DL, Braud BE, Booth JH, Graves DE, Heinle JS, McKenzie ED, et al. Heterotaxy patients with total anomalous pulmonary venous return: improving surgical results. Ann Thorac Surg. 2006;82(5):1621-7; discussion 7-8. | 48 |
| 43 | Seale AN, Carvalho JS, Gardiner HM, Mellander M, Roughton M, Simpson J, et al. Total anomalous pulmonary venous connection: impact of prenatal diagnosis. Ultrasound Obstet Gynecol. 2012;40(3):310-8. | 47 |
| 44 | Harris GB, Neuhauser EB, Giedion A. Total anomalous pulmonary venous return below the diaphragm. Am J Roentgenol Radium Ther Nucl Med. 1960;84:436-41. | 47 |
| 45 | Seale AN, Uemura H, Webber SA, Partridge J, Roughton M, Ho SY, et al. Total anomalous pulmonary venous connection: outcome of postoperative pulmonary venous obstruction. J Thorac Cardiovasc Surg. 2013;145(5):1255-62. | 46 |
| 46 | Cinquetti R, Badi I, Campione M, Bortoletto E, Chiesa G, Parolini C, et al. Transcriptional deregulation and a missense mutation define ANKRD1 as a candidate gene for total anomalous pulmonary venous return. Hum Mutat. 2008;29(4):468-74. | 46 |
| 47 | Smallhorn JF, Sutherland GR, Tommasini G, Hunter S, Anderson RH, Macartney FJ. Assessment of total anomalous pulmonary venous connection by two-dimensional echocardiography. Br Heart J. 1981;46(6):613-23. | 46 |
| 48 | Caldarone CA, Najm HK, Kadletz M, Smallhorn JF, Freedom RM, Williams WG, et al. Surgical management of total anomalous pulmonary venous drainage: impact of coexisting cardiac anomalies. Ann Thorac Surg. 1998;66(5):1521-6. | 45 |
| 49 | Behrendt DM, Aberdeen E, Waterson DJ, Bonham-Carter RE. Total anomalous pulmonary venous drainage in infants. I. Clinical and hemodynamic findings, methods, and results of operation in 37 cases. Circulation. 1972;46(2):347-56. | 44 |
| 50 | Honjo O, Atlin CR, Hamilton BC, Al-Radi O, Viola N, Coles JG, et al. Primary sutureless repair for infants with mixed total anomalous pulmonary venous drainage. Ann Thorac Surg. 2010;90(3):862-8. | 43 |
| 51 | Smallhorn JF, Freedom RM. Pulsed Doppler echocardiography in the preoperative evaluation of total anomalous pulmonary venous connection. J Am Coll Cardiol. 1986;8(6):1413-20. | 43 |
| 52 | Shi X, Huang T, Wang J, Liang Y, Gu C, Xu Y, et al. Next-generation sequencing identifies novel genes with rare variants in total anomalous pulmonary venous connection. EBioMedicine. 2018;38:217-27. | 42 |
| 53 | Lodge AJ, Rychik J, Nicolson SC, Ittenbach RF, Spray TL, Gaynor JW. Improving outcomes in functional single ventricle and total anomalous pulmonary venous connection. Ann Thorac Surg. 2004;78(5):1688-95. | 41 |
| 54 | Newfeld EA, Wilson A, Paul MH, Reisch JS. Pulmonary vascular disease in total anomalous pulmonary venous drainage. Circulation. 1980;61(1):103-9. | 41 |
| 55 | Kauffman SL, Ores CN, Andersen DH. Two cases of total anomalous pulmonary venous return of the supracardiac type with stenosis simulating infradiaphragmatic drainage. Circulation. 1962;25:376-82. | 41 |
| 56 | Heinemann MK, Hanley FL, Van Praagh S, Fenton KN, Jonas RA, Mayer JE, Jr., et al. Total anomalous pulmonary venous drainage in newborns with visceral heterotaxy. Ann Thorac Surg. 1994;57(1):88-91. | 40 |
| 57 | Husain SA, Maldonado E, Rasch D, Michalek J, Taylor R, Curzon C, et al. Total anomalous pulmonary venous connection: factors associated with mortality and recurrent pulmonary venous obstruction. Ann Thorac Surg. 2012;94(3):825-31; discussion 31-2. | 39 |
| 58 | Chowdhury UK, Airan B, Malhotra A, Bisoi AK, Saxena A, Kothari SS, et al. Mixed total anomalous pulmonary venous connection: anatomic variations, surgical approach, techniques, and results. J Thorac Cardiovasc Surg. 2008;135(1):106-16, 16 e1-5. | 39 |
| 59 | Ganesan S, Brook MM, Silverman NH, Moon-Grady AJ. Prenatal findings in total anomalous pulmonary venous return: a diagnostic road map starts with obstetric screening views. J Ultrasound Med. 2014;33(7):1193-207. | 38 |
| 60 | Lupinetti FM, Kulik TJ, Beekman RH, 3rd, Crowley DC, Bove EL. Correction of total anomalous pulmonary venous connection in infancy. J Thorac Cardiovasc Surg. 1993;106(5):880-5. | 38 |
| 61 | Freedom RM, Olley PM, Coceani F, Rowe RD. The prostaglandin challenge. Test to unmask obstructed total anomalous pulmonary venous connections in asplenia syndrome. Br Heart J. 1978;40(1):91-4. | 38 |
| 62 | Lucas RV, Jr., Adams P, Jr., Anderson RC, Varco RL, Edwards JE, Lester RG. Total anomalous pulmonary venous connection to the portal venous system: a cause of pulmonary venous obstruction. Am J Roentgenol Radium Ther Nucl Med. 1961;86:561-75. | 38 |
| 63 | Shumacker HB, King H. A modified procedure for complete repair of total anomalous pulmonary venous drainage. Surg Gynecol Obstet; 1961,112:763-765. | 38 |
| 64 | Parr GV, Kirklin JW, Pacifico AD, Blackstone EH, Lauridsen P. Cardiac performance in infants after repair of total anomalous pulmonary venous connection. Ann Thorac Surg. 1974;17(6):561-73. | 37 |
| 65 | Van Praagh R, Harken AH, Delisle G, Ando M, Gross RE. Total anomalous pulmonary venous drainage to the coronary sinus. A revised procedure for its correction. J Thorac Cardiovasc Surg. 1972;64(1):132-5. | 37 |
| 66 | Mustard WT, Keith JD, Trusler GA. Two-stage correction for total anomalous pulmonary venous drainage in childhood. J Thorac Cardiovasc Surg. 1962;44:477-85. | 37 |
| 67 | Volpe P, Campobasso G, De Robertis V, Di Paolo S, Caruso G, Stanziano A, et al. Two- and four-dimensional echocardiography with B-flow imaging and spatiotemporal image correlation in prenatal diagnosis of isolated total anomalous pulmonary venous connection. Ultrasound Obstet Gynecol. 2007;30(6):830-7. | 36 |
| 68 | Cope JT, Banks D, McDaniel NL, Shockey KS, Nolan SP, Kron IL. Is vertical vein ligation necessary in repair of total anomalous pulmonary venous connection? Ann Thorac Surg. 1997;64(1):23-8; discussion 9. | 36 |
| 69 | Chin AJ, Sanders SP, Sherman F, Lang P, Norwood WI, Castaneda AR. Accuracy of subcostal two-dimensional echocardiography in prospective diagnosis of total anomalous pulmonary venous connection. Am Heart J. 1987;113(5):1153-9. | 36 |
| 70 | Gersony WM, Bowman O, Jr., Steeg CN, Hayes CJ, Jesse MJ, Malm JR. Management of total anomalous pulmonary venous drainage in early infancy. Circulation. 1971;43(5 Suppl):I19-24. | 36 |
| 71 | Burroughs JT, Kirklin JW. Complete surgical correction of total anomalous pulmonary venous connection; report of three cases. Proc Staff Meet Mayo Clin. 1956;31(6):182-8. | 36 |
| 72 | Edwards JE, Helmholz HF, Jr. A classification of total anomalous pulmonary venous connection based on developmental considerations. Proc Staff Meet Mayo Clin. 1956;31(6):151-60. | 36 |
| 73 | Oh KH, Choo KS, Lim SJ, Lee HD, Park JA, Jo MJ, et al. Multidetector CT evaluation of total anomalous pulmonary venous connections: comparison with echocardiography. Pediatr Radiol. 2009;39(9):950-4. | 35 |
| 74 | Carter RE, Capriles M, Noe Y. Total anomalous pulmonary venous drainage. A clinical and anatomical study of 75 children. Br Heart J. 1969;31(1):45-51. | 35 |
| 75 | Johnson AL, Wiglesworth FW, Dunbar JS, Siddoo S, Grajo M. Infradiaphragmatic total anomalous pulmonary venous connection. Circulation. 1958;17(3):340-47. | 35 |
| 76 | St Louis JD, Harvey BA, Menk JS, Raghuveer G, O'Brien JE, Jr., Bryant R, 3rd, et al. Repair of "simple" total anomalous pulmonary venous connection: a review from the Pediatric Cardiac Care Consortium. Ann Thorac Surg. 2012;94(1):133-7; discussion 7-8. | 34 |
| 77 | Nakayama Y, Hiramatsu T, Iwata Y, Okamura T, Konuma T, Matsumura G, et al. Surgical results for functional univentricular heart with total anomalous pulmonary venous connection over a 25-year experience. Ann Thorac Surg. 2012;93(2):606-13. | 34 |
| 78 | Huhta JC, Gutgesell HP, Nihill MR. Cross sectional echocardiographic diagnosis of total anomalous pulmonary venous connection. Br Heart J. 1985;53(5):525-34. | 34 |
| 79 | Parsons HG, Purdy A, Jessup B. Anomalies of the pulmonary veins and their surgical significance; report of three cases of total anomalous pulmonary venous return. Pediatrics. 1952;9(2):152-66. | 34 |
| 80 | Khan MS, Bryant R, 3rd, Kim SH, Hill KD, Jacobs JP, Jacobs ML, et al. Contemporary outcomes of surgical repair of total anomalous pulmonary venous connection in patients with heterotaxy syndrome. Ann Thorac Surg. 2015;99(6):2134-9; discussion 9-40. | 33 |
| 81 | Raisher BD, Grant JW, Martin TC, Strauss AW, Spray TL. Complete repair of total anomalous pulmonary venous connection in infancy. J Thorac Cardiovasc Surg. 1992;104(2):443-8. | 33 |
| 82 | Haworth SG. Total anomalous pulmonary venous return. Prenatal damage to pulmonary vascular bed and extrapulmonary veins. Br Heart J. 1982;48(6):513-24. | 32 |
| 83 | Laux D, Fermont L, Bajolle F, Boudjemline Y, Stirnemann J, Bonnet D. Prenatal diagnosis of isolated total anomalous pulmonary venous connection: a series of 10 cases. Ultrasound Obstet Gynecol. 2013;41(3):291-7. | 31 |
| 84 | van de Wal HJ, Hamilton DI, Godman MJ, Harinck E, Lacquet LK, van Oort A. Pulmonary venous obstruction following correction for total anomalous pulmonary venous drainage: a challenge. Eur J Cardiothorac Surg. 1992;6(10):545-9. | 31 |
| 85 | Yamaki S, Tsunemoto M, Shimada M, Ishizawa R, Endo M, Nakayama S, et al. Quantitative analysis of pulmonary vascular disease in total anomalous pulmonary venous connection in sixty infants. J Thorac Cardiovasc Surg. 1992;104(3):728-35. | 31 |
| 86 | Lamb RK, Qureshi SA, Wilkinson JL, Arnold R, West CR, Hamilton DI. Total anomalous pulmonary venous drainage. Seventeen-year surgical experience. J Thorac Cardiovasc Surg. 1988;96(3):368-75. | 31 |
| 87 | Lincoln CR, Rigby ML, Mercanti C, Al-Fagih M, Joseph MC, Miller GA, et al. Surgical risk factors in total anomalous pulmonary venous connection. Am J Cardiol. 1988;61(8):608-11. | 31 |
| 88 | Sinzobahamvya N, Arenz C, Brecher AM, Blaschczok HC, Urban AE. Early and long-term results for correction of total anomalous pulmonary venous drainage (TAPVD) in neonates and infants. Eur J Cardiothorac Surg. 1996;10(6):433-8. | 30 |
| 89 | Choe YH, Lee HJ, Kim HS, Ko JK, Kim JE, Han JJ. MRI of total anomalous pulmonary venous connections. J Comput Assist Tomogr. 1994;18(2):243-9. | 30 |
| 90 | Yee ES, Turley K, Hsieh WR, Ebert PA. Infant total anomalous pulmonary venous connection: factors influencing timing of presentation and operative outcome. Circulation. 1987;76(3 Pt 2):III83-7. | 30 |
| 91 | Cobanoglu A, Menashe VD. Total anomalous pulmonary venous connection in neonates and young infants: repair in the current era. Ann Thorac Surg. 1993;55(1):43-8; discussion 8-9. | 29 |
| 92 | Mathew R, Thilenius OG, Replogle RL, Arcilla RA. Cardiac function in total anomalous pulmonary venous return before and after surgery. Circulation. 1977;55(2):361-70. | 29 |
| 93 | Tucker BL, Lindesmith GG, Stiles QR, Meyer BW. The superior approach for correction of the supracardiac type of total anomalous pulmonary venous return. Ann Thorac Surg. 1976;22(4):374-7. | 29 |
| 94 | Hoashi T, Kagisaki K, Oda T, Kitano M, Kurosaki K, Shiraishi I, et al. Long-term results of treatments for functional single ventricle associated with extracardiac type total anomalous pulmonary venous connection. Eur J Cardiothorac Surg. 2013;43(5):965-70. | 28 |
| 95 | Wilson WR, Jr., Ilbawi MN, DeLeon SY, Quinones JA, Arcilla RA, Sulayman RF, et al. Technical modifications for improved results in total anomalous pulmonary venous drainage. J Thorac Cardiovasc Surg. 1992;103(5):861-70; discussion 70-1. | 28 |
| 96 | Aziz KU, Paul MH, Bharati S, Lev M, Shannon K. Echocardiographic features of total anomalous pulmonary venous drainage into the coronary sinus. Am J Cardiol. 1978;42(1):108-13. | 28 |
| 97 | Chowdhury UK, Subramaniam KG, Joshi K, Varshney S, Kumar G, Singh R, et al. Rechanneling of total anomalous pulmonary venous connection with or without vertical vein ligation: results and guidelines for candidate selection. J Thorac Cardiovasc Surg. 2007;133(5):1286-94, 94 e1-4. | 27 |
| 98 | el-Said G, Mullins CE, and McNamara DG. Management of total anomalous pulmonary venous return. Circulation. 1972;45(6):1240-50. | 27 |
| 99 | Burchell HB. Total anomalous pulmonary venous drainage: clinical and physiologic patterns. Proc Staff Meet Mayo Clin. 1956;31(6):161-7. | 27 |
| 100 | Serraf A, Bruniaux J, Lacour-Gayet F, Chambran P, Binet JP, Lecronier G, et al. Obstructed total anomalous pulmonary venous return. Toward neutralization of a major risk factor. J Thorac Cardiovasc Surg. 1991;101(4):601-6. | 26 |
